# Photoswitching alters fluorescence readout of jGCaMP8 Ca^2+^ indicators tethered to Orai1 channels

**DOI:** 10.1073/pnas.2309328120

**Published:** 2023-09-20

**Authors:** Joseph L. Dynes, Andriy V. Yeromin, Michael D. Cahalan

**Affiliations:** ^a^Department of Physiology and Biophysics, University of California, Irvine, CA 92697; ^b^Institute for Immunology, University of California, Irvine, CA 92697

**Keywords:** Ca^2+^ signaling, genetically encoded indicator, jGCaMP8f, photoswitching

## Abstract

We used electrophysiology and Ca^2+^ channel tethering to evaluate the performance of jGCaMP8 genetically encoded Ca^2+^ indicators (GECIs). Orai1 Ca^2+^ channel–jGCaMP8 fusions were transfected into HEK 293A cells and jGCaMP8 fluorescence responses recorded by simultaneous total internal reflection fluorescence microscopy and whole-cell patch clamp electrophysiology. Noninactivating currents from the Orai1 Y80E mutant provided a steady flux of Ca^2+^ controlled on a millisecond time scale by step changes in membrane potential. Test pulses to −100 mV produced Orai1 Y80E-jGCaMP8f fluorescence traces that unexpectedly declined by ~50% over 100 ms before reaching a stable plateau. Testing of Orai1-jGCaMP8f using unroofed cells further demonstrated that rapid and partial fluorescence inactivation is a property of the indicator itself, rather than channel function. Photoinactivation spontaneously recovered over 5 min in the dark, and recovery was accelerated in the absence of Ca^2+^. Mutational analysis of residues near the tripeptide fluorophore of jGCaMP8f pointed to a mechanism: Q69M/C70V greatly increased (~90%) photoinactivation, reminiscent of fluorescent protein fluorophore cis-trans photoswitching. Indeed, 405-nm illumination of jGCaMP8f or 8m/8s/6f led to immediate photorecovery, and simultaneous illumination with 405 and 488-nm light blocked photoinactivation. Subsequent mutagenesis produced a variant, V203Y, that lacks photoinactivation but largely preserves the desirable properties of jGCaMP8f. Our results point to caution in interpreting rapidly changing Ca^2+^ signals using jGCaMP8 and earlier series GECIs, suggest strategies to avoid photoswitching, and serve as a starting point to produce more photostable, and thus more accurate, GECI derivatives.

## Significance

Fluorescent genetically encoded calcium indicators (GECIs) are used to observe intracellular Ca^2+^ signaling, including Ca^2+^ spikes that accompany neuronal firing. Here, we use electrophysiology and Ca^2+^ channel tethering to evaluate a faster generation of GECI, jGCaMP8, capable of resolving Ca^2+^ signals on a millisecond timescale. Our experiments reveal breakdowns in jGCaMP8 accuracy: jGCaMP8 fluorescence rapidly declines even when Ca^2+^ levels are stable. Uncovering the mechanism of jGCaMP8 photoinactivation, using in situ testing, led to the identification of a jGCaMP8f mutant lacking photoinactivation and strategies to detect and counteract inactivation using 405-nm light. Our results point to caution in interpreting rapidly changing Ca^2+^ signals using jGCaMP8 and earlier-series GECIs and provide an alternative GECI for high-speed imaging of Ca^2+^.

Calcium ions (Ca^2+^) bind to and regulate numerous protein-based molecular machines in eukaryotic cells (1, 2). The activity of these Ca^2+^-dependent proteins is held in check by Ca^2+^ pumps, exchangers, and buffers, until triggered by Ca^2+^ flux through ion channels into the cytosol. Genetically encoded Ca^2+^ indicators (GECIs) have revolutionized our view of Ca^2+^ dynamics and function within living cells, in part due to their ease of genetic targeting (3, 4). GECIs, like most genetically encoded sensors, use fluorescent proteins (FPs) derived from *Aequorea victoria* green fluorescent protein (avGFP) or similar proteins (5, 6). Each FP consists of an 11-stranded beta-barrel of ~240 amino acids with an autocatalytically formed tripeptide fluorophore at its center (7–10). Single FP sensors, as opposed to fluorescence resonance energy transfer (FRET)-based sensors, use structural variants of GFP—either circularly permuted GFPs (cpGFPs), in which the beta-strands of GFP are reordered while retaining protein fluorescence (5, 11); or FPs in which a binding domain has been inserted into the FP beta-barrel. The cpGFPs exhibit decreased folding stability and increased fluorophore protonation which can be reversed by forced association of the N and C ends of the protein; this property has been exploited to make cpGFP-based sensors (5, 11, 12). Most single FP GECIs follow the same basic design, consisting of a tripartite fusion protein with cpGFP flanked by a C-terminal calmodulin (CaM), which provides Ca^2+^ binding, and an N-terminal CaM target peptide (13). Ca^2+^-dependent association of CaM and its target peptide promotes closure of the protein’s beta-barrel, protonation of the fluorophore, and increased fluorescence emission.

Over the years, mutagenesis has improved GECI properties, including brightness, on and off rates, dynamic range, and Ca^2+^ affinity (5). For the widely used GCaMP 6 and 7 probes, the primary driver for optimization has been detection of neuronal action potentials, and both probes were developed using high throughput screening in neurons (14, 15). Ca^2+^ influx through voltage-gated Ca^2+^ channels accompanies the action potential in many neuronal cell types; in this way, Ca^2+^ has served as a surrogate for changes in membrane potential that accompany neuronal activity. GCaMP6 and 7, and other GECIs, have met with great success in monitoring patterns of neuronal activity underlying static representations of information in the brain, but with fluorescence rise and fall times near 100 ms or more, sensitive detection of ongoing millisecond-scale firing of individual neurons has been out of reach (16–19). Beyond neurons, these GCaMPs have found wide use in monitoring Ca^2+^ signaling in cells throughout the body (3, 20–23). GECI performance needs to be stable, repeatable, and well characterized for accurate Ca^2+^ measurements, which are ultimately required to understand and quantitatively model upstream activation and downstream decoding of Ca^2+^ signals.

Recently, a faster generation of GCaMP indicators, the jGCaMP8 series, has been developed (16). By substituting an endothelial nitric oxide synthase CaM target peptide, and careful attention to linker and intersubunit interface residues, indicator rise time has been lowered into the millisecond range while retaining brightness and useful Ca^2+^ affinity. Similar to GCaMP6 and 7, jGCaMP8 comes in multiple versions (fast/medium/slow) distinguished by their fluorescence fall rate; all three have half-rise times below 10 ms. jGCaMP8 indicators enable detection of single action potentials with a high signal to noise ratio at rates up to 50 Hz, resolving neuronal firing on a timescale widely relevant to neural computation. The capabilities of jGCaMP8, combined with remote optical recording of many sites in parallel (16), should reveal insights into neural information processing—from single neurons to multiple brain regions. Moreover, use of jGCaMP8 in electrically inexcitable cell types promises to reveal previously hidden Ca^2+^ dynamics that result from millisecond-scale openings and closings of other Ca^2+^ permeable ion channels (1, 2).

Fusions of GECIs with Ca^2+^ permeable ion channels have been used to optically monitor local Ca^2+^ currents (24–26). We have used this approach to study channel gating and activity in the non-voltage-gated Orai1 Ca^2+^ channels that mediate store-operated Ca^2+^ entry into numerous electrically inexcitable cell types, including T lymphocytes (27, 28). Orai1 channel fusions have been used also for kinetic testing of attached GECI probes (29). Upon whole-cell patch recording, voltage pulses produce step changes in local Ca^2+^ by changing the driving force of Ca^2+^ through Orai1. Here, we use Orai1-jGCaMP8 fusions to evaluate jGCaMP8 performance on a millisecond time scale. These experiments reveal rapid declines in jGCaMP8 fluorescence unrelated to changes in Ca^2+^ concentration. Identification of a photoinactivation mechanism underlying these fluorescence declines, using local in situ testing, enables strategies to detect and counteract this process and led us to identify a jGCaMP8f mutant that lacks photoinactivation. Our results point to caution in interpreting rapidly changing Ca^2+^ signals using jGCaMP8 and earlier series GECIs and provide an alternative GECI for high-speed imaging of Ca^2+^.

## Results

### Electrical and Optical Recording of Orai1-jGCaMP8f.

We sought to take advantage of the rapid response kinetics of jGCaMP8f to measure Orai1 channel activity on a millisecond time scale. DNA encoding jGCaMP8f was inserted downstream of the Orai1 coding sequence, and Orai1-jGCaMP8f fluorescence responses were monitored in human embryonic kidney (HEK) 293A cells transiently cotransfected with mCherry-STIM1. We combined whole-cell patch electrophysiology and total internal reflection fluorescence (TIRF) imaging, as we did previously (29), and delivered test pulses to negative potentials to drive Ca^2+^ through open Orai1-jGCaMP8f channels while simultaneously monitoring Orai1-jGCaMP8f fluorescence and Ca^2+^ current. We refer to this procedure as Patch-TIRF recording. The activator molecule, mCherry-STIM1, was observed via a second fluorescence channel. During whole-cell recording of Orai1 currents, inclusion of IP_3_ (Inositol 1,4,5 trisphosphate) in the patch pipette leads to IP_3_R opening and endoplasmic reticulum (ER) store depletion, which is sensed by STIM1 proteins in the ER.

**Fig. 1.**
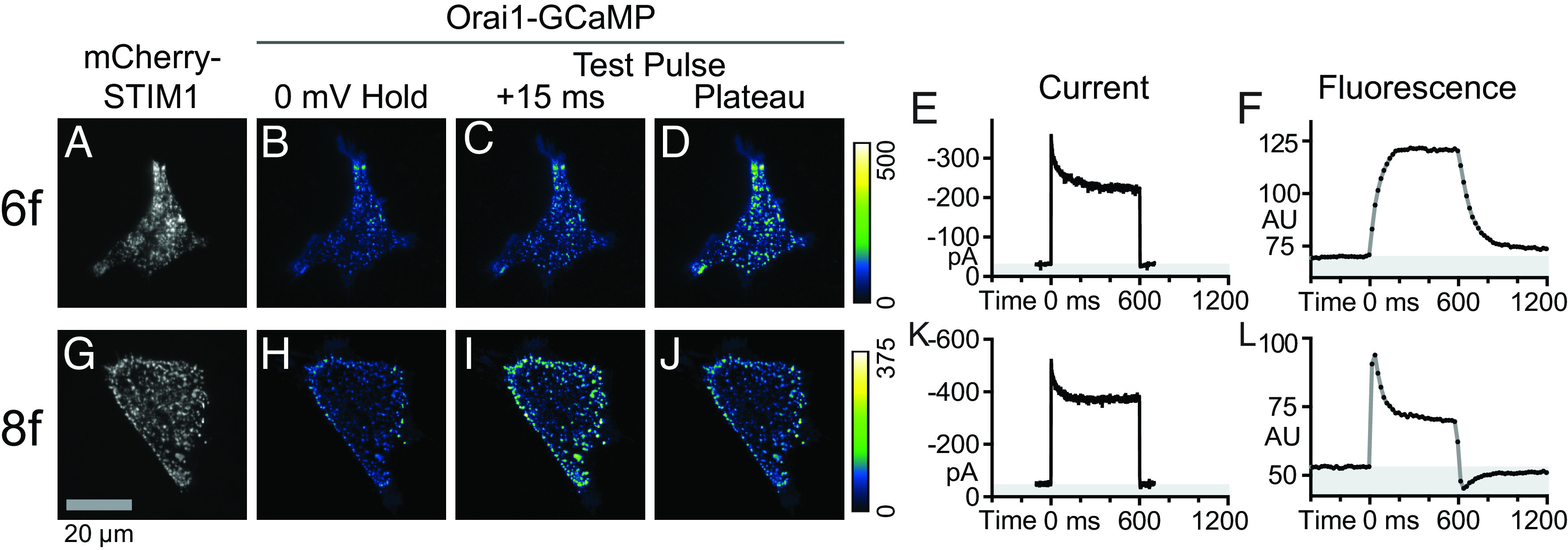
Comparison of Orai1-GCaMP6f and Orai1-jGCaMP8f fluorescence responses to changes in membrane potential. HEK 293A cells cotransfected with mCherry-STIM1 and either Orai1-GCaMP6f (*A*–*F*) or Orai1-jGCaMP8f (*G*–*L*) were simultaneously whole-cell recorded and imaged using TIRFM. (*A* and *B*) TIRF images of mCherry-STIM1 (*A*) and Orai1-GCaMP6f (*B*) at 0-mV holding potential. (*C*–*F*) Response to a 600-ms test pulse to −100 mV. TIRF images of Orai1-GCaMP6f in the first frame (*C*; +15 ms) and plateau (*D*; 500 to 600 ms) of the fluorescence response. Corresponding whole cell current (*E*, not leak subtracted) and Orai1-GCaMP6f fluorescence (*F*) traces. (*G* and *H*) Resting mCherry-STIM1 (*G*) and Orai1-jGCaMP8f (*H*) images. (*I* and *J*) Orai1-jGCaMP8f in the first frame (*I*; +15 ms) and plateau (*J*; 500 to 600 ms) of the fluorescence response to a 600-ms test pulse to −100 mV. (*K* and *L*) Corresponding whole cell current (*K*) and Orai1-jGCaMP8f fluorescence (*L*). Note that the drop in Orai1-jGCaMP8f fluorescence is greater than the drop in Orai1-jGCaMP8f whole cell current. Gray-shaded regions in (*E* and *K*) and (*F* and *L*) indicate current and fluorescence values below baseline, respectively. Data in (*A*–*F*) and (*G*–*L*) are representative of 2 and 15 cells, respectively. AU, arbitrary units of fluorescence intensity.

We began by replicating our previous work (29), using HEK 293A cells cotransfected with Orai1-GCaMP6f and mCherry-STIM1 to document improvements in Orai1-jGCaMP8f kinetics. Activated STIM1 multimerized and translocated to sites in the ER adjacent to the PM (Plasma Membrane) (ER–PM junctions) forming puncta (Fig. 1*A*), as expected. Despite the low level of cytosolic Ca^2+^ at the holding potential (0 mV), Orai1-GCaMP6f was visible in a complementary pattern (Fig. 1*B*). Orai1-GCaMP6f fluorescence rose in the first frame after the onset of a 600-ms test pulse to −100 mV to initiate Ca^2+^ influx (Fig. 1*C*), although first-frame fluorescence was much lower than plateau fluorescence (Fig. 1*D*). Whole-cell current (Fig. 1*E*) showed an immediate rise to peak followed by inactivation over ~100 ms due to fast Ca^2+^-dependent inactivation (fCDI) (30, 31). Orai1-GCaMP6f fluorescence (Fig. 1*F*) rose to a plateau over ~200 ms, which was too slow to reveal fCDI. Orai1-GCaMP6f displayed an inwardly rectified I-V curve (*SI Appendix*, Fig. S1*A*) with a reversal potential near +50 mV, typical for Orai channels (28).

Cotransfected HEK 293A cells showed clustering of mCherry-STIM1 (Fig. 1*G*) and Orai1-jGCaMP8f (Fig. 1*H*) at 0 mV resting potential, like mCherry-STIM1 and Orai1-GCaMP6f cotransfected cells (Fig. 1 *A* and *B*). Also like Orai1-GCaMP6f, Orai1-jGCaMP8f displayed an inwardly rectified I-V curve (*SI Appendix*, Fig. S1*B*) with a reversal potential near +50 mV. However, Orai1-jGCaMP8f demonstrated faster response kinetics compared to Orai1-GCaMP6f. Application of a 600-ms test pulse to −100 mV led to an immediate increase in Orai1-jGCaMP8f fluorescence (Fig. 1*I*), which decayed to a plateau (Fig. 1*J*). Orai1-jGCaMP8f fluorescence declined in a manner similar to Orai1-jGCaMP8f current, but at a slower rate and with a greater degree of inactivation (Fig. 1 *K* and *L*). At the end of the test pulse, Orai1-jGCaMP8f fluorescence immediately dropped below baseline and recovered over ~200 ms. These proof-of-concept experiments show apparently appropriate responses by Orai1-jGCaMP8f during combined whole-cell recording and TIRF imaging. Orai1-jGCaMP8f kinetics are in line with those reported for the unfused jGCaMP8f in neurons (16), given the constraint of our camera acquisition speed. On the face of it, Orai1-jGCaMP8f appears sufficiently fast to track changes in Orai1 channel activity on at least a 20-ms time scale, including changes due to fCDI.

**Fig. 2.**
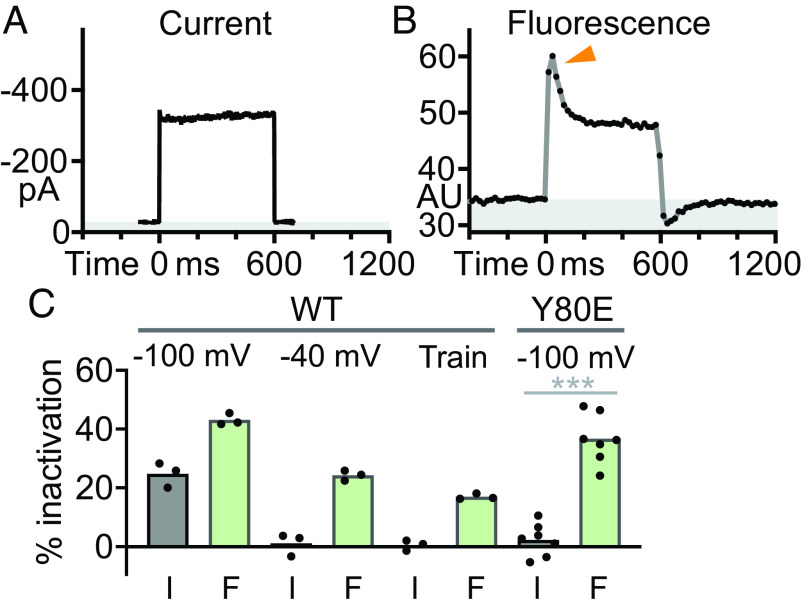
Disparity between Orai1 Y80E-jGCaMP8f whole-cell current and fluorescence responses. HEK 293A cells cotransfected with mCherry-STIM1 and the fCDI mutant Orai1 Y80E-jGCaMP8f were simultaneously whole-cell recorded and imaged using TIRFM. (*A* and *B*) Whole cell current (*A*) and Orai1 Y80E-jGCaMP8f fluorescence (*B*) traces in response to a 600-ms test pulse to −100 mV. Note inactivation apparent in the fluorescence (*B*; orange arrowhead) but not the current (*A*) trace. Gray-shaded regions in (*A*) and (*B*) indicate current and fluorescence values below baseline, respectively. (*C*) Summary graph of inactivation experiments: current (I) and fluorescence (F) inactivation for Orai1-jGCaMP8f in response to single-test pulses to −100 mV and −40 mV and a train of 10-Hz test pulses to −120 mV, and a single-test pulse to −100 mV for Orai1 Y80E-jGCaMP8f. N = 3, 3, 3, and 7 cells, respectively. *** indicates *P* < 0.001, paired *t* test.

To more rigorously evaluate Orai1-jGCaMP8f performance, we used test pulses that induce steady channel currents. These experiments revealed a clear disparity between electrical and optical recordings of Orai1 channel activity (Fig. 2 and *SI Appendix*, Fig. S2). In one approach, we used an Orai1 mutant, Y80E, which lacks fCDI (32), to evoke steady Orai1 channel currents. Cells cotransfected with mCherry-STIM1 and Orai1 Y80E-jGCaMP8f showed little or no current inactivation at −100 mV (Fig. 2*A*); however, the Orai1 Y80E-jGCaMP8f fluorescence response (Fig. 2*B*, arrow) showed a large (~50%) and rapid decline from a peak not present in the current trace (Fig. 2*C*; *P* = 0.0002, paired *t* test). Smaller differences were observed with wild-type Orai1 channels and weak test pulses to −40 mV, a combination which also fails to elicit fCDI. In response to a 600-ms test pulse to −40 mV, current from Orai1-jGCaMP8f (*SI Appendix*, Fig. S2*A*) showed no inactivation, while Orai1-jGCaMP8f fluorescence traces showed a decline following an initial peak (*SI Appendix*, Fig. S2*B*). Another example was seen in responses to trains of test pulses to −120 mV (10 Hz for 1 s). While Orai1-jGCaMP8f current responses were similar in magnitude for every pulse in the train (*SI Appendix*, Fig. S2*C*), the first fluorescence response was larger than subsequent responses (*SI Appendix*, Fig. S2*D*). Taken together (Fig. 2*C*), these data reveal a rapid and partial inactivation phase of Orai1-jGCaMP8f fluorescence that is unrelated to channel activity, and thus unrelated to local [Ca^2+^], which equilibrates on a microsecond time scale (33, 34).

### Photoinactivation and Recovery of jGCaMP8f and Related Ca^2+^ Indicators.

**Fig. 3.**
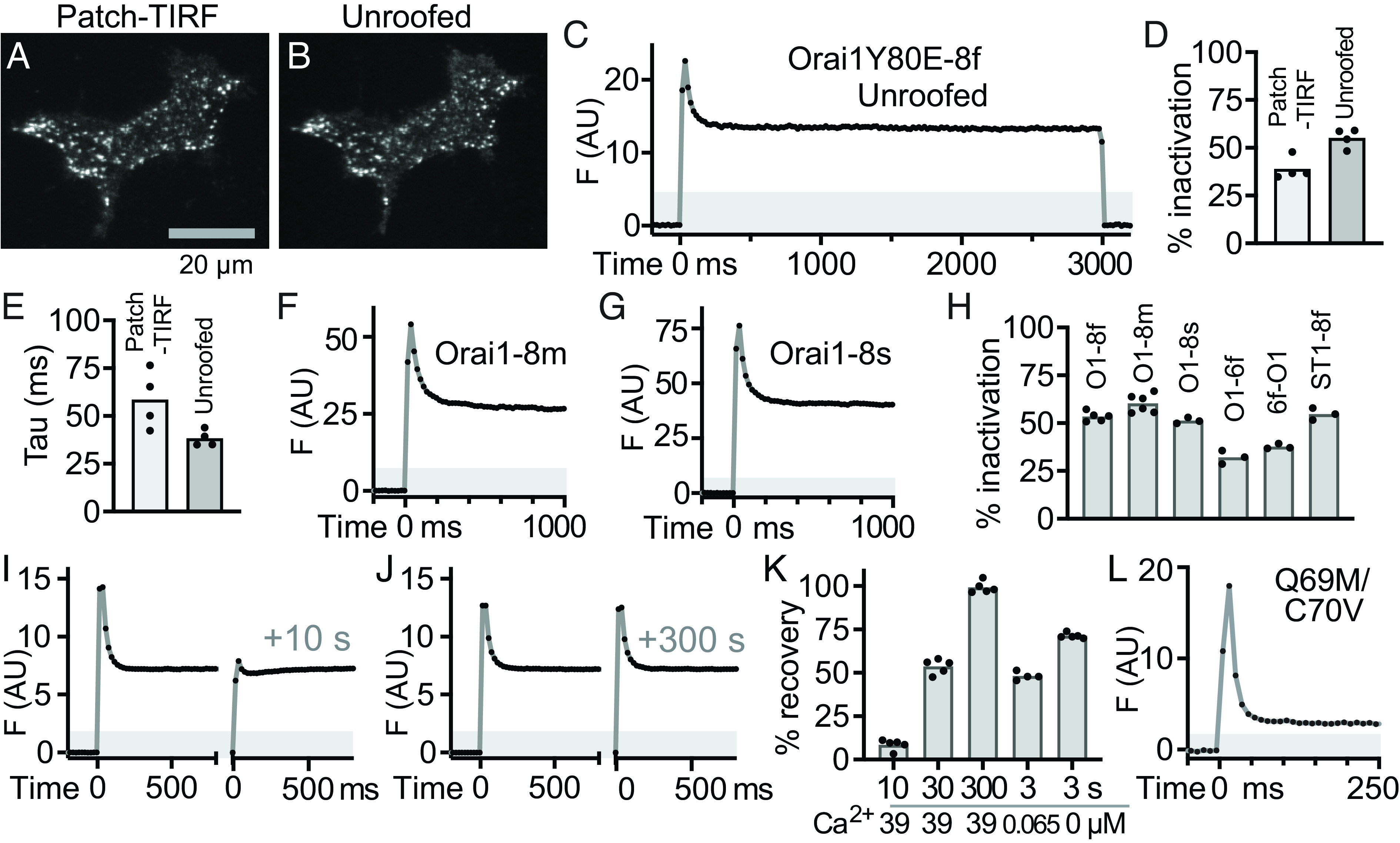
Photoinactivation and recovery of jGCaMP8f and related GECIs in unroofed cells. HEK 293A cells were cotransfected with mCherry-STIM1 and GECI fusion proteins, unroofed, and imaged in situ in 39 µM Ca^2+^ using TIRFM. (*A* and *B*) TIRF images of Orai1 Y80E-jGCaMP8f during whole-cell recording (*A*) and after cell unroofing (*B*). (*C*) Trace of Orai1 Y80E-jGCaMP8f fluorescence from an unroofed cell in which laser illumination was turned on at 0 ms and off at 3,000 ms. (*D* and *E*) Comparison of Orai1 Y80E-jGCaMP8f fluorescence photoinactivation between combined whole-cell recording/TIRF imaging and unroofed cells, measured by percent inactivation (*D*) and exponential decay constant Tau (*E*). N = 3 cells for Patch-TIRF N = 3 cells for unroofed cells. (*F* and *G*) Photoinactivation of Orai1-jGCaMP8m (*F*) and Orai1-jGCaMP8s (*G*). (*H*) Summary graph including Orai1-jGCaMP8 fusions and related proteins Orai1-GCaMP6f, GCaMP6f-Orai1, and STIM1-jGCaMP8f. N = 5, 6, 3, 3, 3, and 3 cells respectively. (*I* and *J*) Recovery of jGCaMP8f from photoinactivation. Pairs of Orai1-jGCaMP8f photoinactivation traces from the same unroofed cell separated by 10 s (*I*) and 300 s (*J*). (*K*) Summary graph of time and [Ca^2+^] dependence of recovery. (*L*) Photoinactivation trace of the mutant jGCaMP8f Q69M/C70V showing an increased rate (note expanded time scale) and extent of inactivation. Gray-shaded regions in (*C*, *F*, *G*, *I*, *J*, and *L*) indicate fluorescence values below a 0 mM Ca^2+^+EGTA baseline.

The disconnect between Orai1-jGCaMP8f current and fluorescence might be a consequence of rapid and partial photoinactivation of the jGCaMP8f protein itself and unrelated to changes in channel activity, channel structure, or local molecular environment. At this point, we use the term “photoinactivation” to denote the rapid decline in jGCaMP8f fluorescence; we reserve “photobleaching” for the irreversible loss of fluorescence due to photodestruction of fluorescent molecules. To gain access to jGCaMP8f responses independent of channel function, we turned to imaging unroofed cells (Fig. 3). After whole cell recording, the upper surface and cell body were removed (“unroofed”) (29, 35), leaving the basal PM in contact with the coverslip largely intact and the inner surface of the membrane accessible to changes in solution composition. The localization of jGCaMP8f during whole cell recording (Fig. 3*A*) was largely unchanged by unroofing (Fig. 3*B*). For experiments with unroofed cells, TIRF illumination was started while the camera was already recording images, to avoid missing changes in fluorescence that might occur between shutter opening and the start of image acquisition. Ca^2+^ was held at a concentration (39 µM) that saturates binding sites on jGCaMP8f (K_d_ = 325 nM) (16) and leads to a maximally fluorescent configuration of the protein. Orai1-Y80E-jGCaMP8f fluorescence in unroofed cells declined rapidly upon TIRF illumination (Fig. 3*C*), reaching a photostable plateau by ~200 ms. This pattern of initial peak, rapid decline, and stable plateau was similar in patched and unroofed cells (cf. Figs. 2*B* and 3*C*). Indeed, when Orai1-Y80E-jGCaMP8f cells were illuminated with the same intensity of 488-nm light, percent inactivation (Fig. 3*D*) and exponential decay constants (Fig. 3*E*) were similar between patched and unroofed cells. These experiments show that photoinactivation of jGCaMP8f can account, in large part, for the disparities between whole cell current and fluorescence.

Other GECIs showed photoinactivation in unroofed cells as well. Photoinactivation traces similar to jGCaMP8f were obtained for two other jGCaMP8 series members, 8m and 8s (Fig. 3 *F*–*H*). GCaMP6f also showed photoinactivation (*SI Appendix*, Fig. S3*A*), although to a lesser degree. Photoinactivation was observed when the position of GCaMP6f was moved from the C to the N terminus of Orai1 (GCaMP6f-Orai1; *SI Appendix*, Fig. S3*B*) or when jGCaMP8f was moved to another puncta protein (STIM1-jGCaMP8f; *SI Appendix*, Fig. S3*C*), suggesting that inactivation may not be due to the local molecular environment of the GECI. Moreover, we observed no evidence of photoconversion of jGCaMP8f to a red fluorescent form (*SI Appendix*, Fig. S3*D*). To further ensure that the decay in fluorescence intensity was due to photoinduced inactivation of the GECI, and not some other aspect of experimental design, we varied the illumination intensity during photoinactivation experiments. We found that progressive decreases in illumination intensity produced progressive decreases in the rate of inactivation (*SI Appendix*, Fig. S3 *E*–*G*), indicating that GECI inactivation is a light-dependent process. Taken together, these experiments demonstrate that illumination with 488-nm light induces rapid and partial fluorescence inactivation of GCaMP indicators in the presence of Ca^2+^.

Photoinactivation of jGCaMP8f was not caused by irreversible bleaching, but instead, fluorescence spontaneously recovered over minutes in the absence of illumination at room temperature and saturating Ca^2+^. Recovery was assessed by acquiring a photoinactivation image stream of an unroofed cell cotransfected with Orai1-jGCaMP8f, followed by a second image stream after a defined interval. jGCaMP8f showed little recovery in saturating 39 µM Ca^2+^ when one image stream followed another by 10 s (Fig. 3*I*). Recovery increased to 50% after 30 s (*SI Appendix*, Fig. S3*H*) and was essentially complete by 5 min (Fig. 3*J*). The small degree of recovery over 10 s provided an assay for measuring the Ca^2+^ dependence of recovery. Recovery was accelerated by transiently lowering the Ca^2+^ concentration to 65 nM for 3 s (50% recovery; *SI Appendix*, Fig. S3*I*) and accelerated even more when Ca^2+^ was removed entirely for 3 s (70% recovery; *SI Appendix*, Fig. S3*J*). Taken together, these experiments show that recovery of jGCaMP8f fluorescence depends upon both time and Ca^2+^ concentration (Fig. 3*K*). Moreover, they suggest that photoinactivation is driven by light-induced structural changes within the FP beta barrel of jGCaMP8f, because Ca^2+^removal, which opens the beta barrel, facilitates photorecovery.

Because of the uniquely reactive nature of cysteine residues, we mutated the two cysteines in the FP portion of jGCaMP8f to determine whether they contribute to fluorescence photoinactivation. To simplify comparison with the literature, we retain the avGFP amino acid numbering to describe mutations in the cpGFP-based jGCaMP8f. avGFP contains cysteines at residues 48 and 70, both with inward-facing side chains conserved in jGCaMP8f (16). C70 is proximal to the tripeptide fluorophore derived from amino acids 65 to 67, while C48 is more distal as part of the beta-barrel cap. The mutations C48S and C70V did not appreciably change jGCaMP8f photoinactivation in unroofed cells (*SI Appendix*, Fig. S4 *A* and *B*). However, another mutant from this series, Q69M/C70V, taken from the sequence of the FP T-Sapphire (36), unexpectedly showed more photoinactivation and faster decay (Fig. 3*L* and *SI Appendix*, Fig. S4 *C* and *D*).

### Photoswitching of jGCaMP8f by 405-nm Light.

**Fig. 4.**
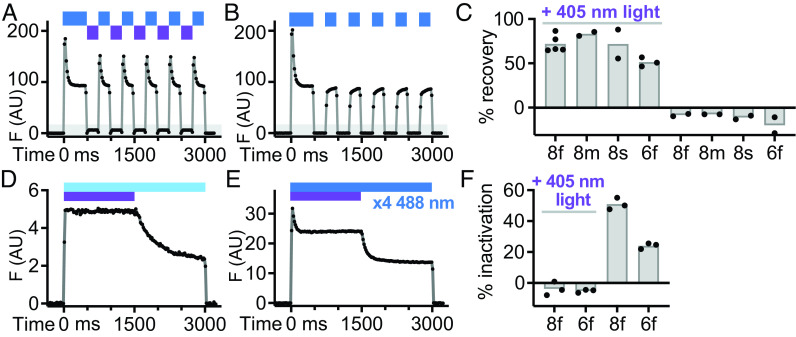
Photoswitching of jGCaMP8f by 405-nm light. (*A*–*F*) Unroofed cells were imaged by TIRFM in 39 µM Ca^2+^. (*A*) Fluorescence trace of Orai1-jGCaMP8f in which illumination alternated between 220 ms light pulses at 488 nm (blue bar) and 405 nm (purple bar). Note the repeated recovery of jGCaMP8f fluorescence after 405-nm illumination. (*B*) Orai1-jGCaMP8f fluorescence trace using the same illumination protocol as in (*A*) except 405-nm light was omitted. (*C*) Summary graph of fluorescence recovery by different GECIs. N = 5, 2, 2, and 3, for 8f, 8m, 8s, and 6f with 405-nm light, and N = 2, 2, 2, and 2 without 405-nm light, respectively. (*D*–*F*) Blocking photoinactivation by 405-nm light at 1:1 (*D*) and 1:4 (*E*) intensity ratios (405:488 nm), with summary graph (*F*; N = 3 for 8f and 6f, respectively). Gray-shaded regions in (*A* and *B*) indicate fluorescence values below a 0 mM Ca^2+^+EGTA baseline.

The rapid decay of the double mutant Q69M/C70V is reminiscent of a class of FPs termed reversibly switchable FPs, which are photoinactivated by the same wavelength of light used for excitation (37). In the case of the GFP-based reversibly switchable FPs, fluorescence can be recovered by illumination with 405-nm light, which is absorbed by the fluorophore and switches it from a trans-neutral-non-fluorescent to a cis-anionic-fluorescent form (38)—also termed a E-Z conformational change. We hypothesized that jGCaMP8f might be an unrecognized reversibly switchable FP, and we tested this possibility using unroofed cells cotransfected with Orai1-jGCaMP8f which were intermittently illuminated with wide-field 405-nm light. Illumination by 405-nm light for 220-ms produced immediate recovery of jGCaMP8f fluorescence after photoinactivation (Fig. 4*A*), which was not observed in control experiments (Fig. 4*B*). In addition, 405-nm light illumination also led to photorecovery of jGCaMP8m, jGCaMP8s, and GCaMP6f fluorescence (Fig. 4*C*).

We next hypothesized that jGCaMP8f photoswitching could be continuously reversed by simultaneous illumination of the sample with 405-nm light, such that jGCaMP8f fluorescence output would show no sign of photoinactivation. To test this hypothesis, we first measured the intensity and spatial profile of the 488-nm TIRF and 405-nm wide-field illumination lasers using fluorescein in solution (*SI Appendix*, *SI Materials and Methods*). When Orai1-jGCaMP8f in unroofed cells was illuminated with a combination of 405- and 488-nm light, photoinactivation was entirely blocked (Fig. 4*D*). Upon shuttering the 405-nm laser, photoinactivation resumed. Decreasing the ratio of 405- to 488-nm light led to partial protection from photoinactivation and a two-step inactivation profile, with one photoinactivation step observed with both lasers and second step with just the 488-nm laser (Fig. 4*E*). GCaMP6f photoinactivation was also entirely suppressed by a combination of 405- and 488-nm light (Fig. 4*F*). Finally, we tested whether jGCaMP8f photoinactivation occurred in live intact cells, and not just in patched cells during whole cell recording and in unroofed cells. HEK 293A cells cotransfected with Orai1-jGCaMP8f and mCherry-STIM1 were treated with 2 µM of the SERCA pump inhibitor thapsigargin in Ringer solution lacking Ca^2+^ and imaged with TIRFM (Total Internal Reflection Fluorescence Microscopy). Upon shift to 2 mM Ca^2+^ Ringer, Orai1-jGCaMP8f fluorescence increased but then showed a typical photoinactivation decline (*SI Appendix*, Fig. S5*A*). Illumination with 405-nm light for 220-ms produced an immediate recovery of Orai1-jGCaMP8f fluorescence (*SI Appendix*, Fig. S5*A*), which was not present in control experiments (*SI Appendix*, Fig. S5 *B* and *C*). Laser scanning confocal microscopy revealed an essentially identical pattern of photoinactivation and 405-nm light–dependent recovery (*SI Appendix*, Fig. S5 *D*–*F*). These experiments show that jGCaMP8f/8m/8s/6f fluorescence is reversibly switchable and that 405-nm light can locally reveal and continuously reverse photoinactivation of the probe.

### jGCaMP8f V203Y Blocks Photoinactivation.

**Fig. 5.**
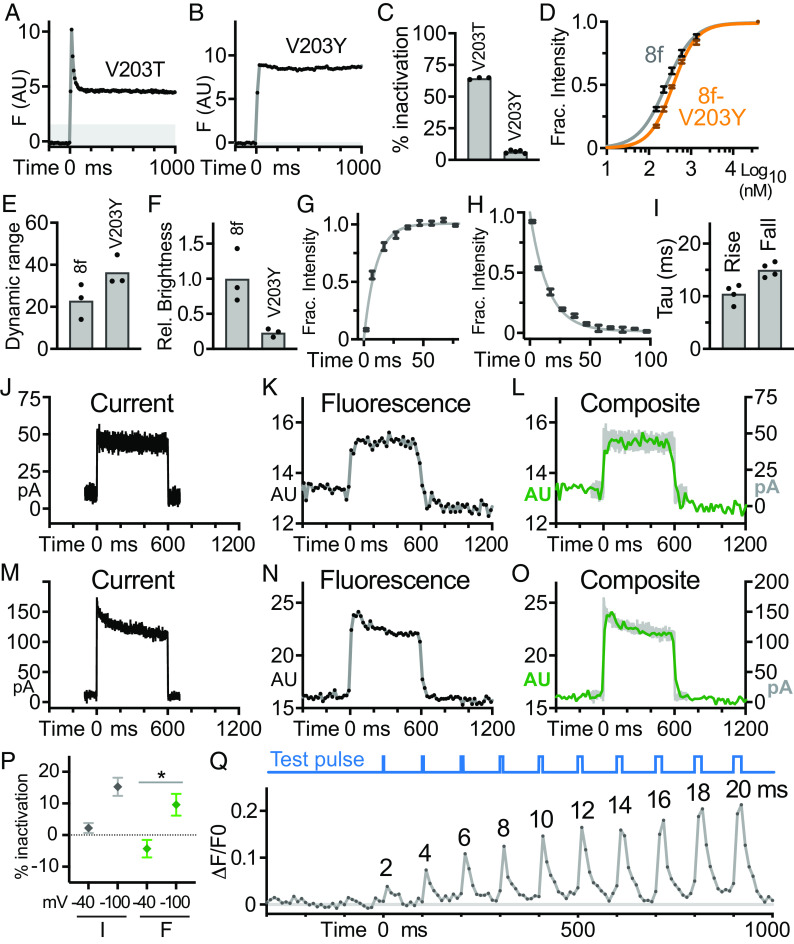
Photoinactivation and response dynamics of jGCaMP8f V203Y. (*A*–*C*) Photoinactivation curves from unroofed cells in 39 µM Ca^2+^ for Orai1-jGCaMP8f V203T (*A*) and Orai1-jGCaMP8f V203Y (*B*) and inactivation graph (*C*). Inactivation of Orai1-jGCaMP8f V203Y was 6.5 ± 0.5% (N = 4 cells); Orai1-jGCaMP8f V203T was 64 ± 0.5 % (N = 5 cells). (*D*) Fluorescence-based Ca^2+^-binding curve for Orai1-j GCaMP8f V203Y (orange, N = 4 cells) and Orai1-jGCaMP8f (gray, N = 5 cells). K_d_ and Hill coefficient values for Orai1-jGCaMP8f V203Y were 370 nM (95% CI 350 to 390) and 1.56 (95% CI 1.45 to 1.68), and for Orai1-jGCaMP8f were 260 nM (95% CI 230 to 280 nM) and 1.33 (95% CI 1.14 to 1.54), respectively. (*E*) Orai1-GECI dynamic range calculated using 0 µM and 39 µM Ca^2+^. (*F*) Orai1-jGCaMP8f V203Y brightness in 39 µM Ca^2+^ relative to Orai1-jGCaMP8f, derived by imaging mCherry-STIM-GECI fusions with 488-nm light. For (*E*) and (*F*), N = 3 and 3 cells, respectively. (*G* and *H*) Rise (*G*) and fall (*H*) plots of Orai1 Y80E-jGCaMP8f V203Y fluorescence in response to test pulses to −100 mV. N = 4 and 4 cells for (*G*) and (*H*), respectively. (*I*) Graph of rise and fall rates. (*J*–*O*) Plots of Orai1-jGCaMP8f V203Y current (*J* and *M*), fluorescence (*K* and *N*), and superimposed current and fluorescence (*L* and *O*) for test pulses to −40 mV, a noninactivating potential (*J*–*L*) and −100 mV, an inactivating potential (*M*–*O*). Traces are representative of N = 7 cells. (*P*) Graph of fluorescence inactivation 50 to 250 ms after the start of test pulses. * indicates *P* < 0.05, N = 7 cells. “I” indicates current, and “F” indicates fluorescence. Relative inactivation (the difference in inactivation at −100 mV and −40 mV) was similar between fluorescence (13.9 ± 4.4%) and current (13.0 ± 3.3%). (*Q*) Orai1 Y80E-jGCaMP8f V203Y fluorescence response to a series of increasing duration test pulses to −100 mV. Test pulse duration is indicated above the plot, with a test pulse schematic above the plot in blue. Representative trace from N = 4 cells. Error bars in (*D*, *G*, *H*, and *P*) are ± SEM.

Understanding that fluorophore photoswitching causes jGCaMP8f photoinactivation allowed us to rationally design mutations to reduce photoinactivation. We noted that many yellow FPs contain a tyrosine residue at amino acid 203 which interacts with a tyrosine in the tripeptide FP fluorophore via pi-stacking (39). We introduced V203Y into jGCaMP8f with the aim of stabilizing the active fluorophore via pi-stacking, thus limiting interconversion to the trans-neutral-non-fluorescent form. For comparison, we made a second substitution at position 203, V203T, which reverted a change originating in GCaMP3 back to the avGFP/EGFP amino acid (40). Photoinactivation remained high in jGCaMP8f V203T (Fig. 5*A*). In contrast, photoinactivation was almost completely blocked in jGCaMP8f V203Y (Fig. 5 *B* and *C*). The loss of photoinactivation was not due to disruption of Ca^2+^ sensing by jGCaMP8f V203Y, as its Ca^2+^ affinity (370 nM) and dynamic range, determined in unroofed cells, were largely unchanged (Fig. 5 *D* and *E*). However, jGCaMP8f V203Y brightness was reduced fourfold (Fig. 5*F*).

These results suggest that jGCaMP8f V203Y could function as an accurate reporter of rapidly changing Ca^2+^ levels, and we assessed jGCaMP8f V203Y dynamics using Patch-TIRF. The rapid rise and fall rates of jGCaMP8f were preserved in jGCaMP8f V203Y (Tau_rise_ and Tau_fall_ of 10 and 15 ms, respectively; Fig. 5 *G*–*I*), which were tested using a fusion to the noninactivating Orai1 mutant Y80E in order to avoid confounding effects of fCDI. As another test of indicator dynamics, we returned to the problem of monitoring fCDI of Orai1 currents. fCDI of Orai1 typically occurs in two phases, fit by exponential decay functions with rate constants of roughly 10 and 100 ms (30, 31). Given our measured jGCaMP8f V203Y rise rate of 10 ms, the first decay phase is too fast to be tracked in its entirety, but the second phase should be easily monitored. Orai1-jGCaMP8f V203Y fluorescence traces largely paralleled the corresponding current traces. (Fig. 5 *J*–*P*). At −40 mV, Orai1-jGCaMP8f V203Y fluorescence rapidly reached a plateau within 50 ms and did not inactivate, whereas at −100 mV, fluorescence reached an initial peak which then declined to a plateau. Inactivation measured by fluorescence was significantly greater at −100 mV than −40 mV (Fig. 5*P*). While fluorescence reported a slightly lower degree of inactivation than current (~5% lower), relative inactivation (the difference in inactivation at −100 mV and −40 mV) was similar. Finally, a series of −100 mV test pulses lasting from 2 ms to 20 ms were delivered to determine the minimum detectable pulse duration. Responses to 4-ms test pulses were easily identified in single traces (Fig. 5*Q*), and even 2-ms pulses were detectable in averaged traces (*SI Appendix*, Fig. S6). Taken together, these results show that photoinactivation can be blocked by probe mutation while preserving many desirable qualities of jGCAMP8f.

## Discussion

We combined patch-clamp electrophysiology, simultaneous TIRFM, direct nanometer linkage to an ion channel Ca^2+^ source, and local in situ testing to evaluate jGCaMP8 performance. The combination of steady Ca^2+^ levels and rapid fluorescence readout allowed us to document pervasive photoinactivation and spontaneous recovery of jGCaMP8 fluorescence. We found that jGCaMP8 photoinactivation was a consequence of fluorophore photoswitching; elucidation of this mechanism enabled strategies to locally detect, counteract, and mutationally suppress it. Our results point to caution in interpreting rapidly changing Ca^2+^ signals using jGCaMP8 and earlier series GECIs and serve as a significant step in producing bright, fast GECI derivatives that are more photostable, and thus more accurate.

Several lines of evidence show that rapid but partial declines in Orai1-jGCaMP8 fluorescence are caused by photoinactivation of the GECI probe, including multiple experimental configurations (Patch-TIRF, unroofed and live cells), multiple GECIs, and multiple fusion protein targets. The existence of a stereotyped recovery and a dependence upon the intensity of 488-nm light further support a defined photoinactivation process. Photoinactivation has been described for regular FPs, most notably the red fluorescent mApple (41). Moreover, photoinactivation and switching have been engineered into GCaMP5G-based sensors for superresolution imaging of Ca^2+^ signaling (42). Some GCaMP indicators display rapid but partial photoinactivation under resting conditions at the start of an imaging session, including preliminary versions of the original GCaMP, as well as GCaMP7c and jGCaMP8m (13, 16). A similar pattern of photoinactivation has been described for another cpGFP-based sensor, the genetically encoded voltage indicator ASAP2 (43). Here, we describe a different pattern of photoinactivation that alters fluorescence readout of activated GECIs in response to Ca^2+^ signals. This activation-dependent process could account for the previously described fluorescence baseline declines of jGCaMP8m, GCaMP7c, and early GCaMPs, if these indicators were substantially Ca^2+^-bound in resting cells (13, 16).

While the extent of jGCaMP8f photoinactivation was consistent in unroofed cells, declines were more variable in Patch-TIRF with noninactivating currents. We attribute this variability to differences in Orai1 channel activity, and thus local [Ca^2+^], caused by different test pulse potentials and different levels of channel activation among transiently transfected cells. We also note that unfused GCaMPs were not tested, as tethering was required for protein retention during whole cell recording and with unroofed cells, but we know of no process by which membrane tethering could convert a GECI into a reversibly switchable FP. Even if photoinactivation were limited to tethered GECIs, this would be a significant limitation, since local, subcellular detection of Ca^2+^ is an important and widely used feature of GECIs (25, 27, 44, 45).

Illumination by 405-nm light promoted immediate recovery of jGCaMP8f fluorescence in both unroofed and live cells, which we attribute to the known absorption of 405-nm light by the trans-neutral form of the fluorophore (37). Moreover, mutation of an amino acid known to interact with the fluorophore (aa 203) prevented photoinactivation and thus photoswitching. Why are jGCaMP8/6 proteins susceptible to photoswitching? Within the FP beta-barrel, the fluorophore is held in position by a network of hydrogen bonds and steric crowding from inward-facing amino acid side chains (9, 10). A prominent source of fluorophore hydrogen bonding, H148, was lost in the widely used aa 149 to 238 + 1 to 144 cpGFP inversion, although in some sensors bonding is re-established via an intermediate water molecule internal to the beta-barrel (5). For jGCaMP8 indicators, a tyrosine in the first EF-hand of calmodulin, Y352 (jGCaMP8f numbering), provides this water-mediated hydrogen bond with the fluorophore (16). Also lost in cpGFP is Y145, which does not form a hydrogen bond with the fluorophore, but instead acts to hold the fluorophore in place, presumably through a combination of steric crowding and Van der Waals forces (46, 47). We speculate that loss of H148 and Y145 decreases the energy required to convert the fluorophore to an inactive form, despite the presence of an alternate bonding network, such as jGCaMP8 Y352. Pi-stacking of the fluorophore by the mutant V203Y likely establishes a more robust fluorophore interaction network and thus stabilizes the active fluorophore.

Our data suggest that photoinactivation via photoswitching may be a widespread issue in sensors based on cpGFPs. However, inactivation may be less obvious in some contexts. For instance, GECIs might recover from photoinactivation between frames when the acquisition interval is long. When the time course of photoinactivation is slow due to low illumination intensity, declines might be misinterpreted as changes in Ca^2+^ channel/pump activity or photobleaching. Similarly, in vitro measurements of GECI performance, using less focused and lower intensity light than standard microscopy, might miss photoinactivation. During high-speed imaging, photoinactivation might be obscured by the slow rise rates of GCaMP6/7 and earlier indicators, or by a narrow focus on event detection (for instance, with action potentials). Now, however, the millisecond scale Ca^2+^ response of jGCaMP8—the key improvement of this series—increases the likelihood that jGCaMP8 experiments will be confounded by photoinactivation and recovery. These processes depend upon a combination of experimenter-chosen illumination parameters (wavelength, intensity, duration, interval) and cellular Ca^2+^ responses (concentration, duration, clearance, interval). The interplay of these factors complicates efforts to predict and numerically compensate for photoinactivation.

We address the problem of GECI photoinactivation in three ways. First, we provide a test to detect when images of Ca^2+^ signals are affected by photoinactivation using intermittent illumination with 405-nm light. Second, we developed a procedure to continuously reverse GECI photoinactivation using simultaneous illumination with 405-nm light, analogous to the partial photoreversion of the voltage indicator ASAP2 with 405-nm light (43). Note that adding 405-nm light may lead to inadvertent excitation of other fluorophores and increased background fluorescence. Combination of GCaMP imaging and optogenetics presents an additional concern. Optogenetic activation using 405-nm light would cause recovery of fluorescence emission from previously inactivated GCaMP fluorophores; resultant photorecovery-based elevations in GCaMP fluorescence may be incorrectly attributed to elevations in subcellular Ca^2+^ in the absence of suitable controls (48, 49). It is also possible that the degree and rate of jGCaMP8 photoswitching may differ between 1- and 2-photon excitation.

Finally, we identified a mutant of jGCaMP8f that lacks photoinactivation, V203Y. jGCaMP8f V203Y preserves desirable characteristics of jGCaMP8f, such as rise and fall rates, Kd, and dynamic range, while allowing photoinactivation to be bypassed without using 405-nm light. The rise and fall rates of jGCaMP8f V203Y appear comparable to values for the parent jGCaMP8f protein, although it is difficult to accurately measure jGCaMP8f rates due to photoinactivation. Photoinactivation inflates rising fluorescence values at the start of a test pulse compared to the plateau and depresses falling fluorescence values at the end of a test pulse with a dip below baseline, in both cases altering rate calculations. Even without further development, jGCaMP8f V203Y is useful for millisecond time scale recording of Ca^2+^ channel activity, and we used it to monitor fCDI of Orai1 channel currents. jGCaMP8f V203Y fluorescence closely tracked test pulse currents, with a lag in fluorescence consistent with our measured rise and fall rates (of 10 and 15 ms, respectively). Measurements of relative channel inactivation (−100 mV vs. −40 mV) with jGCaMP8f V203Y fluorescence showed good agreement with respective measurements using channel current. Single-trial detection of test pulses 4 ms long shows that jGCaMP8f V203Y excels at detecting brief Ca^2+^ transients and expands the temporal range of channel dynamics accessible by GECIs. We note that jGCaMP8f V203Y is dimmer than jGCaMP8f but expect subsequent mutagenesis will increase brightness. Ultimately, jGCaMP8f V203Y narrows the gap in accuracy between optical and electrical recording of rapidly changing Ca^2+^ currents.

## Materials and Methods

Detailed methods are described in *SI Appendix*, *SI Materials and Methods*.

### Cell Culture, Molecular Biology, and Transfections.

HEK 293A cells (Invitrogen) were cultured and transfected as in ref. 29.

### Whole-Cell Recording.

Whole-cell recordings were performed with transfected HEK 293A cells as described previously (29).

### Cell Unroofing.

HEK 293A cells were monitored visually using transmitted light and unroofed by applying pipette pressure, in a procedure modified from ref. 29.

### TIRF Microscopy.

TIRF imaging was performed on an Olympus IX81 inverted microscope with a home-built TIRF illuminator, Olympus 60x 1.45 N.A. PlanApoN TIRF objective, and 488-nm and 561-nm solid-state lasers. Two camera/optical splitters combinations were used: a Photometrics Evolve 512 EMCCD camera with a Photometrics DualView2 2-channel splitter, and a Photometrics Prime 95B sCMOS camera and Cairn Optosplit III 3-channel splitter.

### Photoactivation with 405-nm Light.

Photoactivation was performed with a 405-nm solid state laser, which was directed to the sample from above the focal plane in a wide-field illumination pattern.

### Confocal Microscopy.

Cells were imaged on an Olympus FV3000 laser scanning confocal microscope equipped with a UPLXAPO60XO oil objective (NA 1.42).

### Image Processing, Analysis, and Statistical Testing.

Image processing and measurements were performed using the Fiji implementation of ImageJ2 version 2.3.0/1.53F; intensity calculations were performed in Microsoft Excel version 16.64; and statistical testing, SEM calculation, and graphing were performed using GraphPad Prism version 9.4.1.

## Supplementary Material

Appendix 01 (PDF)

## Data Availability

All study data are included in the article and/or *SI Appendix*.
