# Supplementary material for: Photoswitching alters fluorescence readout of jGCaMP8 Ca2+ indicators tethered to Orai1 channels
Source: Proc Natl Acad Sci U S A. 2023 Sep 20;120(39):e2309328120. doi: 10.1073/pnas.2309328120 (PMC10523504; doi:10.1073/pnas.2309328120)
Supplement: Supplementary file 1 — Appendix 01 (PDF) [file pnas.2309328120.sapp.pdf]

Supporting Information for:

Photoswitching alters fluorescence readout of jGCaMP8 Ca^{2+} indicators tethered to Orai1 channels

Joseph L. Dynes[a,1], Andriy V. Yeromin[a,1], and Michael D. Cahalan[a,b,2]

[a]Department of Physiology and Biophysics and [b]Institute for Immunology, University of California, Irvine, CA 92697, USA

[1]Contributed equally to this work

[2]Corresponding Author:

Michael D. Cahalan

Email: mcahalan@uci.edu

This PDF file includes:

Supporting text
Figures S1 to S6
Tables S1 to S2
SI References

Supporting Information Text

SI Materials and Methods

Cells and Transfection

Cell culture and transfection protocols have been described previously (1). Briefly, human embryonic kidney (HEK) 293A cells (ThermoFisher Scientific) were grown in DMEM medium (Lonza) supplemented with 10% fetal bovine serum (Omega Scientific) and 2 mM L-glutamine (Sigma-Aldrich), referred to here as complete DMEM. Cells were transfected in 6-well plates at 80-90% confluency using Lipofectamine 2000 (ThermoFisher Scientific) according to manufacturer instructions. A total of 2 µg of DNA at a 3:1 ratio of STIM1 to Orai1 was used for transfection. One day after transfection, cells were dissociated from the culture surface with TrypLE Express (ThermoFisher), spun down, resuspended in complete DMEM, plated onto poly L-lysine-coated coverslips (>300K MW, 0.1 mg/ml in water; Sigma-Aldrich), and incubated overnight before imaging and whole-cell recording.

Molecular Biology and DNA Cloning

Plasmids created in this study, as well as other plasmids used, are listed in Supplemental Table S1; DNA oligonucleotide primers used in plasmid construction are listed in Supplemental Table S2. All plasmids produced using polymerase chain reaction (PCR) and site-directed mutagenesis were confirmed by DNA sequencing. Plasmids encoding jGCaMP8 series genetically encoded Ca^{2+} indicators (GECIs) were obtained from Addgene: pGP-CMV-jGCaMP8f (#162373), pGP-CMV-jGCaMP8m (#162372), pGP-CMV-jGCaMP8s (#162371). For Orai1-jGCaMP8 fusions, GECIs were PCR amplified using N- and C-terminal primers (G8FORC and G8REVC) and ligated into plasmid Orai1-EGFP (2) AgeI–NotI, replacing the EGFP gene. To create Orai1 Y80E-jGCaMP8f, first Orai1 Y80E was PCR amplified from GGECO1.2-Orai1 Y80E using N- and C-terminal primers (Orai1FORC and Orai1REVC) and ligated into EGFP-N1 XhoI–EcoRI to create Orai1 Y80E-EGFP. Next, jGCaMP8f was cut out of Orai1-jGCaMP8f AgeI–NotI and ligated into Orai1 Y80E-EGFP, replacing the EGFP

gene. To create Orai1 Y80E-GCaMP8f V203Y, Orai1 Y80E was cut out of Orai1 Y80E-jGCaMP8f using NheI-BamHI and ligated into Orai-jGCaMP8f V203Y (see below), replacing the wild-type Orai1 gene.

Site-directed mutations were made in two ways: by PCR amplification with in vitro recombination via InFusion cloning (TaKaRa) and by traditional site-directed mutagenesis (QuickChange II XL kit, Aligent). jGCaMP8f mutations C48S and Q69M/C70V were created by InFusion cloning using procedures provided by the manufacturer. Primers incorporating jGCaMP8f mutations C48S and Q69M/C70V, along with N- and C-terminal primers, were used to PCR amplify the jGCaMP8f gene. Since the nucleotide sequences encoding C48 and Q69/C70 are close together, the 5' ends of the respective primers were designed to overlap, enabling in vitro recombination and InFusion cloning. For jGCaMP8f C48S, primer pairs IF-Fwd1 + IF-C48SRev1 and IF-Fwd2 + IF-Rev2 were used, and for jGCaMP8f Q69M/C70V, primer pairs IF-Fwd1 + IF-Rev1 and IF-Q69M/C70VFwd2 + IF-Rev2 were used. Orai1-jGCaMP8f was the recipient plasmid vector after digestion with MluI and NotI to remove parent jGCaMP8f indicator. jGCaMP8f mutations C70V, V203T, and V203Y were created in the plasmid Orai1-jGCaMP8f via site-directed mutagenesis using procedures provided by the manufacturer and primers listed in Supplemental Table S2.

The double-label STIM1 constructs mCherry-STIM1-jGCaMP8f and mCherry-STIM1-jGCaMP8f V203Y used to measure GECI relative brightness were created as follows. First, the human STIM1 coding region was amplified via PCR from the plasmid pcDNA3(+)zeo-hSTIM1 (3), using S1FORC2 and S1REVC2 primers and ligated EcoRI-BamHI into the plasmid EGFP-N1 (Clontech), producing STIM1-EGFP. Next, jGCaMP8f was cut out of the plasmid Orai1-jGCaMP8f AgeI-NotI and ligated into STIM1-EGFP, replacing the EGFP gene and producing STIM1-jGCaMP8f. Finally, mCherry-STIM1-EGFP and mCherry-STIM1-jGCaMP8f were created by cutting mCherry and a portion of the STIM1 gene from mCherry-STIM1 (4), NheI-BspEI and ligating into STIM1-EGFP and STIM1-jGCaMP8f, respectively. mCherry-STIM1-jGCaMP8f V203Y was created by replacing the wild-type Orai1 gene from Orai1-jGCaMP8f V203Y with mCherry-STIM1 from mCherry-STIM1-eGFP using NheI-BamHI

Whole-cell Recording and Solutions

Electrophysiological procedures are similar to those described (1). Whole-cell recordings were acquired using an EPC9 patch clamp amplifier (HEKA) with a 5 kHz sampling rate and digitally filtered to 1-2 kHz for analysis and display. Pipette (2-4 MΩ) and cell capacitances were compensated completely, and series resistance was compensated by 80%, by EPC9 circuitry. Membrane potential was corrected for the liquid junction potential (-13 mV) between the pipette and bath solutions. The external solution contained (in mM) 151.5 NaCl, 4.5 KCl, 2 $CaCl_2$, 1 $MgCl_2$, 10 glucose, 10 HEPES, 8 sucrose; pH was adjusted to 7.4 by NaOH with a final osmolality of 325 mOsm. The pipette solution contained (in mM) 122 CsAsp, 12 Cs_4EGTA, 2 CsCl, 10 $MgGluconate_2$, 2 Na_2ATP, 15 HEPES, 20 μM inositol 1,4,5 trisphosphate (IP_3); pH was adjusted to 7.2 by CsOH with a final osmolality of 301 mOsm. After break-in, membrane potential was held at 0 mV. Voltage ramps from -80 to +60 mV were alternated with 220-ms pulses to -80 mV every 2 s to monitor Orai1 current development and cell health. Electrophysiology data were analyzed using the computer programs Pulse (HEKA), Excel (Microsoft), and Origin (OriginLab).

TIRF Microscopy

TIRF imaging was performed on an Olympus IX81 inverted microscope with a home-built objective-based TIRF illumination system, as described (2). The system was controlled by Metamorph 7 software (Molecular Devices). TIRF illumination lasers, a 68 mW 488-nm solid-state laser (Coherent) and a 48 mW 561-nm solid-state laser (Lasos), were introduced into the optical pathway by way of a micrometer-positioned 5-mm mirror-coated prism positioned near the back focal plane of the objective. Lasers were mechanically shuttered using Uniblitz VS25 shutters (Vincent Associates) driven by a Model 10-2 multifunctional filter/shutter controller (Sutter). Laser power was attenuated using a combination of a continuously variable and fixed neutral density filters.

Two configurations of the imaging system were used, differing in the camera and optical splitter, but in each case using the same Olympus 60x 1.45 N.A. PlanApoN TIRF objective. Initial experiments

used a Photometrics DualView2 two-channel image splitter containing a Chroma T565lpxr dichroic mirror and Semrock 520/28 (green) and 624/40 (red) emission filters, and a Photometrics Evolve 512 electron multiplying charge-coupled device (EMCCD) camera. 16-bit images were acquired using an EM camera gain of 130, which corresponds to ~20 AU/photon (AU = Arbitrary Units of fluorescence intensity). Using the 60x TIRF objective, a pixel corresponded to a 267 nm square with this camera. Image streams were acquired episodically at 50 frames/s using reduced-height (<256 pixels) regions of interest (ROI). Subsequent experiments used a Cairn OptoSplit III three-channel image splitter containing Chroma ZT543rdc and ZT594rdc dichroic mirrors and Semrock 520/28 (green), 575/15 + LP02-561RU (orange), and 640/40 (red) emission filters and a Photometrics Prime 95B scientific complementary metal-oxide-semiconductor (sCMOS) camera. 12-bit images were acquired using the "high sensitivity" mode, which corresponded to ~2 AU/photon. Using the 60x TIRF objective, a pixel corresponded to a 183 nm square with this camera. Image streams were acquired episodically at 50-200 frames/s using reduced-height (400 pixels) ROIs, with a corresponding frame read time of ~4 ms. Fluorescence intensity values from the Evolve 512 camera were normalized to those from the Prime 95B camera by dividing by 20 to account for a 10-fold difference in photon-AU mapping and a 2-fold difference in pixel area.

The activity of Orai1-jGCaMP8f and similar fusion proteins were monitored typically in the green channel and mCherry-STIM1 in the red channel, which were acquired simultaneously using only the 488-nm laser to ensure uniform registration of excitation light to both fluorophores. 488-nm laser input power was typically attenuated to 3 mW using a 1.3 optical density (OD) neutral density filter. TIRF illumination intensity at the sample was determined by first imaging the laser illumination pattern using fluorescein in solution and then measuring the light reflected off the cover slip by the TIRF beam using a laser power meter; average illumination intensity of a centered 25x40 μm ROI was 8 W/cm^2. Laser power was measured before every experiment with a Thorlabs PM400 optical power meter and S121C photodiode head.

Laser scanning confocal microscopy

HEK 293A cells were imaged with an Olympus FV3000 laser scanning confocal microscope with inverted IXplore Pro microscope frame using an Olympus UPLXAPO60XO oil objective (NA 1.42). Puncta formation was documented using 488-nm (Orai1-jGCaMP8f) and 561-nm (mCherry-STIM1) laser illumination. Photo-switching tests were performed with 488-nm and 405-nm lasers set at 4% laser power, which corresponded to 0.107 and 0.128 mW at the sample, respectively, measured with a Thorlabs PM400 optical power meter and S121C photodiode head. 512x512 pixels images were recorded at 4 frames per s with a pixel dwell time of 500 µs. Green channel fluorescence was detected using a GaAsP photomultiplier tube with a 500-540 nm spectral bandpass.

Combined Whole-cell Recording and TIRF Imaging

Whole-cell recording and TIRF imaging, either singly or simultaneously (Patch-TIRF), were performed at room temperature. The flux of Ca^{2+} through Orai1-GECI channels, and thus the local [Ca^{2+}] at the indicator, was controlled by step changes in membrane potential (test pulses). Test pulses to -100 mV with wild-type Orai1 channel fusions produced inactivating currents, and test pulses to -40 mV with wild-type Orai1 channel fusions or -100 mV with Orai1 Y80E fusions produced non-inactivating currents. 300 and 600 ms duration test pulses were used to evaluate Orai1-GECI fluorescence responses, which occurred 500 to 1000 ms after the start of camera acquisition.

In initial experiments, image streams were synchronized with test pulses and whole-cell recordings using Transistor-Transistor Logic (TTL) pulses sent from the patch-clamp amplifier to an analogue-to-digital converter computer card (IOtech) controlled by Metamorph 7 software during image acquisition, as described (1). Subsequent experiments used direct triggering of the Prime 95B camera by the patch-clamp amplifier via TTL pulses. This configuration was used for high-speed and high temporal accuracy measurements, such as GECI rise/fall times.

Cell Unroofing

HEK 293A cells were unroofed using pipette pressure, different from mechanical unroofing used in our previous study (1). Cells were unroofed either at the end of a Patch-TIRF session, or in the absence of voltage-controlled image acquisition protocols, when Orai1 current reached a stable plateau. In either case, cells were unroofed after depletion of ER Ca^{2+} stores by diffusion of IP_3 from the patch pipette. Pipette pressure was increased using a syringe under visual control until the plasma membrane (PM) on the upper surface of the cell ruptured. The pipette was gently withdrawn without disturbing the coverslip-attached basal PM, which retained the same distribution of STIM1-Orai1 puncta. Unroofed cells were stable for more than one hour at room temperature.

Image Processing and Fluorescence Measurements

Image processing and measurements were performed using the Fiji implementation (version 2.3.0/1.53F) (5) of the freely available image processing program, ImageJ, produced by the Research Service Branch of the National Institutes of Mental Health and Neurological Disorders and Stroke (6). Fluorescence intensity calculations were performed in Microsoft Excel version 16.64 and graphs and plots were created in Prism 9.4.1 (GraphPad Software). Cell footprint and adjacent background ROIs were drawn manually for measuring fluorescence intensity.

Measurements of Photoinactivation and Recovery

Photo-inactivation of Orai1-jGCaMP8f fusions during whole-cell recording was measured using steady or repetitive channel currents produced in three ways: (1) a mutant Orai1 channel fusion which lacks fCDI (Y80E) with test pulses to -100 mV, (2) a wild-type Orai1 channel fusion with test pulses to -40 mV, and (3) a wild-type Orai1 channel fusion with 10-Hz trains of 10 50-ms test pulses to -120 mV. For conditions (1) and (2), peak fluorescence was measured in the second frame after the start of the test pulse, since first -frame fluorescence was more strongly affected by probe rise time. Plateau values were set as the average of frames from T=+220 to +280 ms. Resting fluorescence at 0 mV, defined as the average of frames from T=-200 to -20 ms before the test pulse, was subtracted from peak and

plateau values, and fractional inactivation calculated by first dividing adjusted plateau fluorescence by adjusted peak fluorescence and subtracting the result from 1. For trains of pulses, peak fluorescence was calculated as the average of the first two frames, since rise time was faster with test pulses to -120 mV. Inactivated peak fluorescence values were calculated as the average peak fluorescence for pulses 2 to 4, and fractional inactivation was calculated from resting fluorescence subtracted values as for single test pulses.

For unroofed cells, photo-inactivation experiments were performed by initiating stream acquisition by the camera and then opening the laser shutters, to avoid missing changes in fluorescence that might occur between shutter opening and the start of image acquisition. Unless specified, unroofed cells were imaged in Calcium Calibration Solution (Invitrogen): a single frame in 0 µM Ca^{2+} + 10 mM EGTA and image streams in 39 µM Ca^{2+}. Peak fluorescence was measured in the second frame after shutter opening, and plateau fluorescence was an average of frames from T=+410 to +490 ms, except for direct comparisons to patched cells, when an average of frames from T=+220 to +280 ms was used. Fluorescence in 0 µM Ca^{2+} + 10 mM EGTA was used as a baseline, which was subtracted from peak and plateau values to calculate fractional inactivation.

For photorecovery experiments, pairs of 3 s stream acquisitions were separated by 10 s (start-to-start). To measure fractional photorecovery, fluorescence was normalized to the plateau fluorescence for each image stream. Photorecovery was calculated by dividing the peak from the second image stream by the peak from the first image stream. Each image stream pair was separated from other image stream pairs by at least five minutes to allow for full photorecovery. For experiments to assess the Ca^{2+} dependence on recovery, unroofed cells were perfused locally with 39 µM Ca^{2+} with a switch to 0 µM Ca^{2+} + 10 mM EGTA or 0.065 µM Ca^{2+} for 3 s between pairs of image streams.

Testing jGCaMP8f for Photoconversion

HEK 293A cells cotransfected with Orai1-jGCaMP8f and myc-STIM1 were unroofed and TIRF imaged in situ in Calcium Calibration Buffer with 39 µM Ca^{2+}. Three-channel (green/orange/red) imaging was

performed using the Prime 95B camera and Optosplit III image splitter. Illumination was alternated between 561-nm light, to detect fluorescence from orange and red fluorescent forms of jGCaMP8f, if they exist, and 488-nm light, to excite the jGCaMP8f probe, induce photo-inactivation, and monitor the time course of photoinactivation and recovery. The first 500-ms 488-nm light pulse starting at T=0 ms induced jGCaMP8f photoinactivation, and the second 500-ms 488-nm light pulse starting at T=+1000 ms was used to confirm that the probe remained in a photoinactivated state throughout the interval of T=+500 to +1000 ms. Fluorescence from any photoinduced orange or red fluorescent form present was calculated separately for each channel by subtracting pre-photoinactivated jGCaMP8f fluorescence (T=-500 to 0 ms) from the post-photoinactivated jGCaMP8f fluorescence (T=+500 to +1000 ms).

Photoactivation using 405-nm Light

Photoactivation was accomplished using a 5-mW 405-nm laser module (Qiaoba) mounted horizontally at the end of a steel rod mounted above the microscope stage on a micromanipulator. The beam was directed downward towards the sample using a right angle prism. Laser output was controlled using a Model 505 laser diode driver (Newport) and measured using a laser power meter; 3 mW was used for photoactivation. Illumination intensity at the sample was determined by imaging the laser illumination pattern with a 4x objective using fluorescein in solution; average illumination intensity of a centered 25x40 µm ROI was 0.2 W/cm^2. Light pulse triggering was controlled and synchronized using Metamorph 7 software via a USB-6501 digital input-output module (National Instruments) and TTL input to the laser power supply.

Measurement of Orai1-GCaMP K_d, Dynamic Range, and Relative Brightness

Orai1-GCaMP K_d was measured from TIRF images obtained upon perfusing buffered Ca^{2+} solutions at a range of Ca^{2+} concentrations across unroofed cells cotransfected with an Orai1-GCaMP fusion and mCherry-STIM1, as described (1). K_d values and Hill coefficients were calculated using Prism 9 (GraphPad Software). Unroofed cells cotransfected with Orai1-GCaMP fusions and myc-STIM1 were

used to derive indicator dynamic range, which was calculated by dividing fluorescence in 39 µM Ca^{2+} by fluorescence in 0 µM Ca^{2+} + 10 mM EGTA after subtracting background fluorescence. Unroofed cells cotransfected with HA-Orai1 and double-label mCherry-STIM1-GCaMP fusions were used to calculate indicator relative brightness. Bleed-through of GCaMP fluorescence into the red channel was determined using unroofed cells cotransfected with Orai1-GCaMP and myc-STIM1. For a given mCherry-STIM1-GCaMP fusion, GCaMP brightness relative to mCherry was calculated by dividing GCaMP fluorescence from mCherry fluorescence after subtracting background fluorescence. The resulting relative brightness calculations were normalized to 1 for mCherry-STIM1-jGCaMP8f. Both GCaMP and mCherry fluorescence were excited by the 488-nm laser only to ensure uniform registration of excitation light to both fluorophores.

Measurement of jGCaMP8f V203Y Kinetics

Cells were cotransfected with Orai1 Y80E-jGCaMP8f V203Y and mCherry-STIM1, and jGCaMP8f V203Y fluorescence responses in Patch-TIRF were measured using test pulses to -100 mV, as described (1). Fusion to the Orai1-jGCaMP8f Y80E mutant was used to avoid confounding effects of fCDI. Images were acquired at 100 frames/s, and the start time of each frame was increased by 2 ms to account for the rolling readout of the sCMOS camera, given a cell centered in the image frame and a 4-ms frame read time. Prism 9 software was used for non-linear curve fitting of normalized traces, rise/fall rate calculations, and plotting. For experiments to detect brief events, a series of brief test pulses were delivered at a rate of one per 100 ms. The duration of test pulses to -100 mV ranged from 2 to 20 ms, with each pulse 2 ms longer than the previous one. Traces were baseline subtracted using fluorescence values from an image stream without a test pulse. For plotting the average fluorescence response by Z-score, fluorescence responses were normalized to the pre-pulse baseline and average peak value in response to test pulses of 16, 18, and 20 ms in duration. The standard deviation of the background was measured from fluorescence values at T=-300 to -10 ms before the start of the test pulse series.

SI Figures

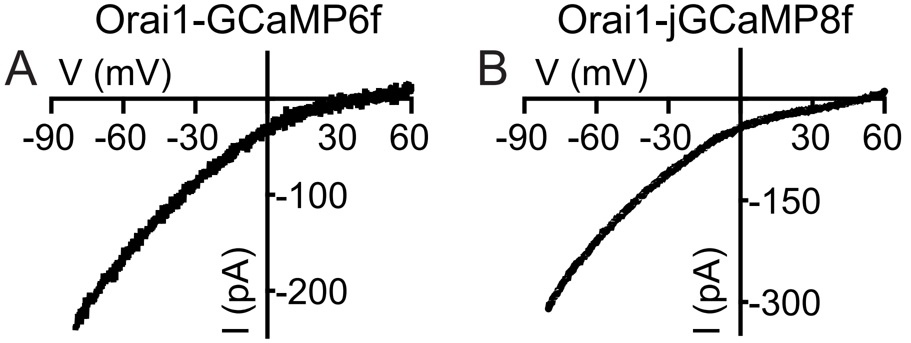

Fig. S1. I-V curves of transfected HEK cells during Patch-TIRF. (A and B) I-V curves for HEK 293A cells cotransfected with mCherry-STIM1 and either Orai-GCaMP6f (A) and Orai-jGCaMP8f (B). Representative of N=2 and 15 cells for (A) and (B) respectively.

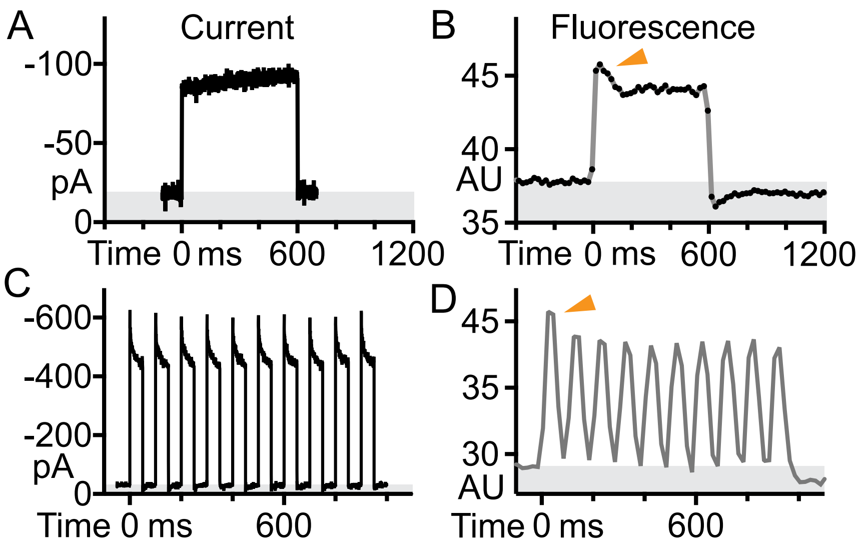

Fig. S2. Disparity between Orai1-jGCaMP8f whole-cell current and fluorescence responses. (A-D) HEK 293A cells cotransfected with mCherry-STIM1 and Orai1-jGCaMP8f were simultaneously whole-cell recorded and imaged using TIRFM. (A and B) Whole-cell current (A) and Orai1-jGCaMP8f fluorescence (B) traces in response to a 600-ms test pulse to -40 mV, a membrane potential that does not elicit fCDI. Note the inactivation apparent in the fluorescence (B; orange arrowhead) but not the current (A) trace. (C and D) Whole-cell current (C) and Orai1-jGCaMP8f fluorescence (D) traces in response to a train of 10-Hz test pulses to -120 mV. Note that the fluorescence response (D) to the first pulse (orange arrowhead) is larger than subsequent pulses. Gray shaded region in (A and C) and (B and D) indicate current and fluorescence values below baseline, respectively. N= 3 and 5 cells for (A and B) and (C and D), respectively.

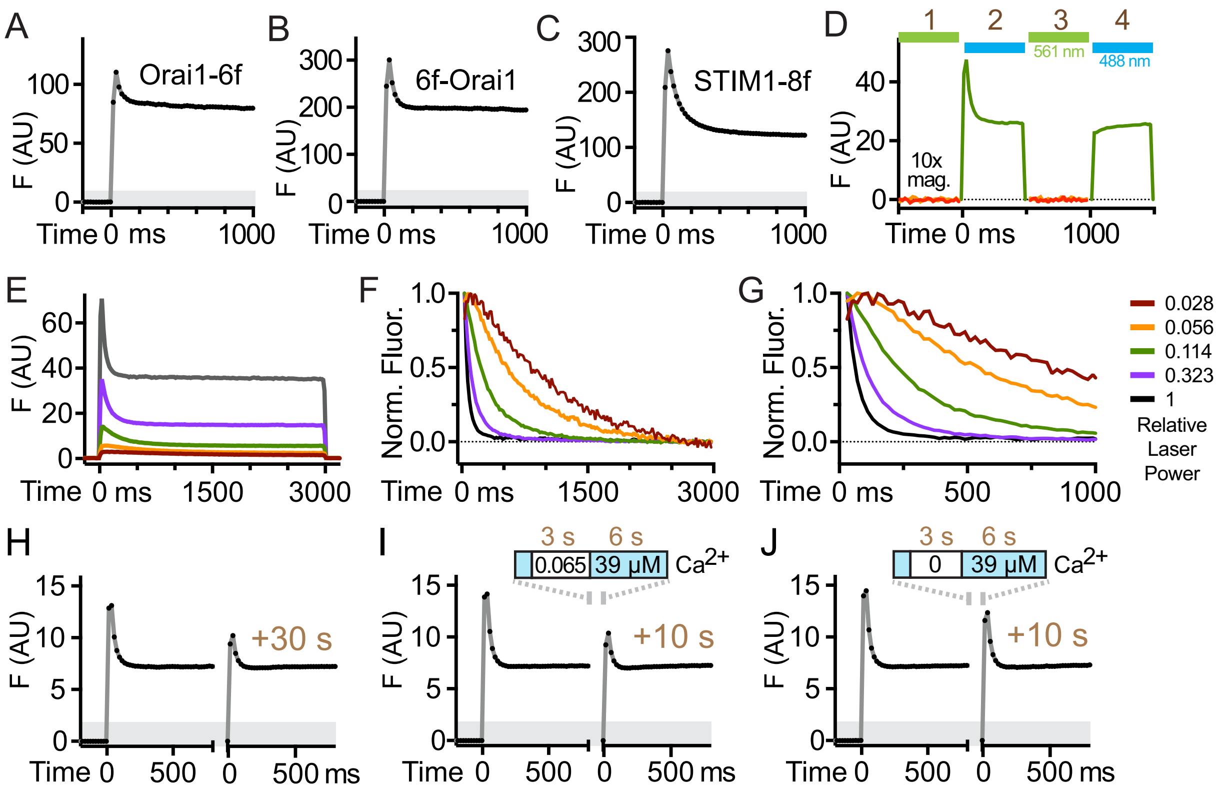

Fig. S3. Photoinactivation and recovery of GECI fusion proteins. HEK 293A cells were cotransfected with mCherry-STIM1 and GECI fusion proteins, unroofed, and imaged in situ in 39 μM Ca^{2+} using TIRFM. (A-C) Traces of Orai1-GCaMP6f (A), GCaMP6f-Orai1 (B), and STIM1-jGCaMP8f (C) fluorescence in which laser illumination was turned on at 0 ms. Traces are representative of N=3, 3, and 3 cells for (A), (B), and (C), respectively. (D) HEK 293A cells were cotransfected with Orai1-jGCaMP8f and myc-STIM1, unroofed, and imaged in situ in 39 μM Ca^{2+} using TIRFM. Illumination by 561 nm (green bar) and 488 nm (blue bar) light was alternated, and corresponding background-subtracted red, orange, and green channel fluorescence are plotted. Red and orange channel fluorescence was multiplied by 10 before plotting to enhance visibility. Note that the red and orange channel fluorescence does not increase after Orai1-jGCaMP8f photo-inactivation. Traces are representative of N=3 cells. (E-G) Photoinactivation of Orai1-jGCaMP8f in unroofed cells using a range of laser illumination powers. Raw fluorescence traces (E), and fluorescence traces normalized to the

peak and plateau (3000 ms) of the response (F and G), were used to visualize photoinactivation kinetics. Traces are representative of 3 cells. (H) Pair of Orai1-jGCaMP8f traces from the same unroofed cell separated by 30 s. Traces are representative of N=5 cells. (I and J) Pairs of Orai1-jGCaMP8f traces separated by 10 s except Ca^{2+} was briefly lowered by perfusion of a 65 nM Ca^{2+} solution (I) or a 10 mM EGTA containing solution lacking Ca^{2+} (J) for 3 s. Traces are representative of N= 4 and 4 cells for (I) and (J), respectively.

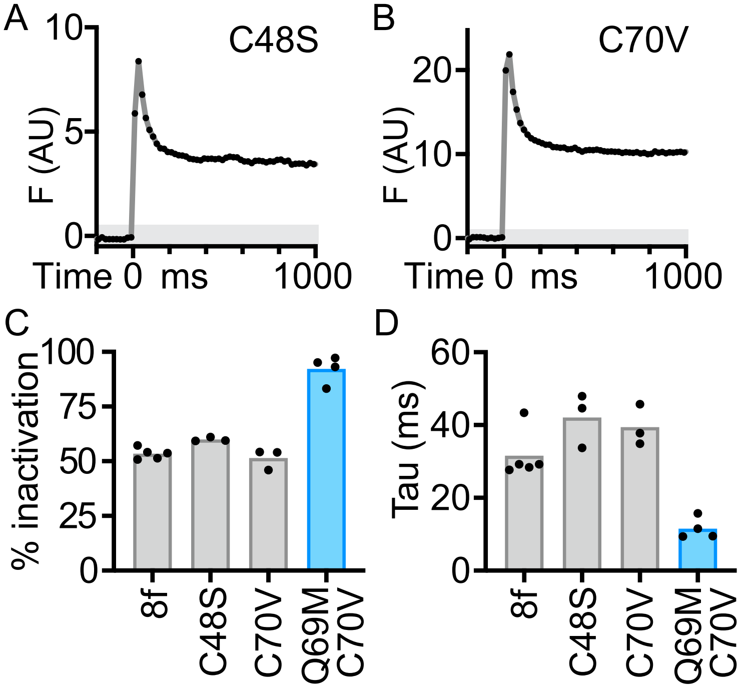

Fig. S4. Fluorescence of jGCaMP8f cysteine mutants. Unroofed cells were imaged by TIRFM in 39 μM Ca^{2+}, with 488-nm illumination starting at T=0 ms. (A and B) Fluorescence traces of Orai1-jGCaMP8f cysteine mutants. (D and E) Comparison of fluorescence declines measured by percent inactivation (C) and exponential decay rate constant Tau (D). N=3 and 3 cells for (A) and (B), respectively, and N=5 and 4 cells for parent jGCaMP8f (8f) and double mutant GECI (Q69M/C70V). Gray shaded region in (A and B) indicate fluorescence values below a 0 mM Ca^{2+}+EGTA baseline.

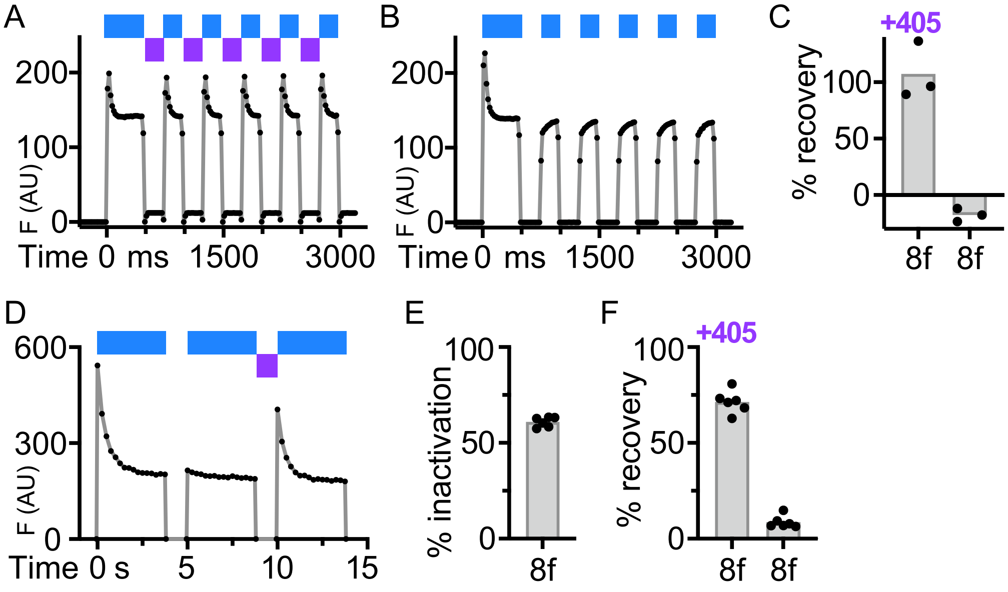

Fig. S5. Photo-switching of jGCaMP8f in live cells. Live intact HEK 293A cells cotransfected with Orai1-jGCaMP8f and mCherry-STIM1 were imaged in Ringer solution containing 2 mM Ca^{2+} after treatment with thapsigargin. (A and B) TIRFM fluorescence traces of Orai1-jGCaMP8f in which illumination alternated between 220-ms light pulses at 488 nm (blue bar) and either (A) 405 nm (purple bar) or (B) no light pulse. Note the repeated recovery of jGCaMP8f fluorescence after 405 nm illumination. (C) Summary graph of fluorescence recovery, N=3 cells. (D) Confocal microscope fluorescence trace of Orai1-jGCaMP8f in which laser scanning illumination is alternated between 4 s with 488-nm laser (blue bar) and 1 s with either no laser or 405-nm laser (purple bar). (E and F) Summary graphs of fluorescence inactivation (E) and recovery (F), N=6 cells.

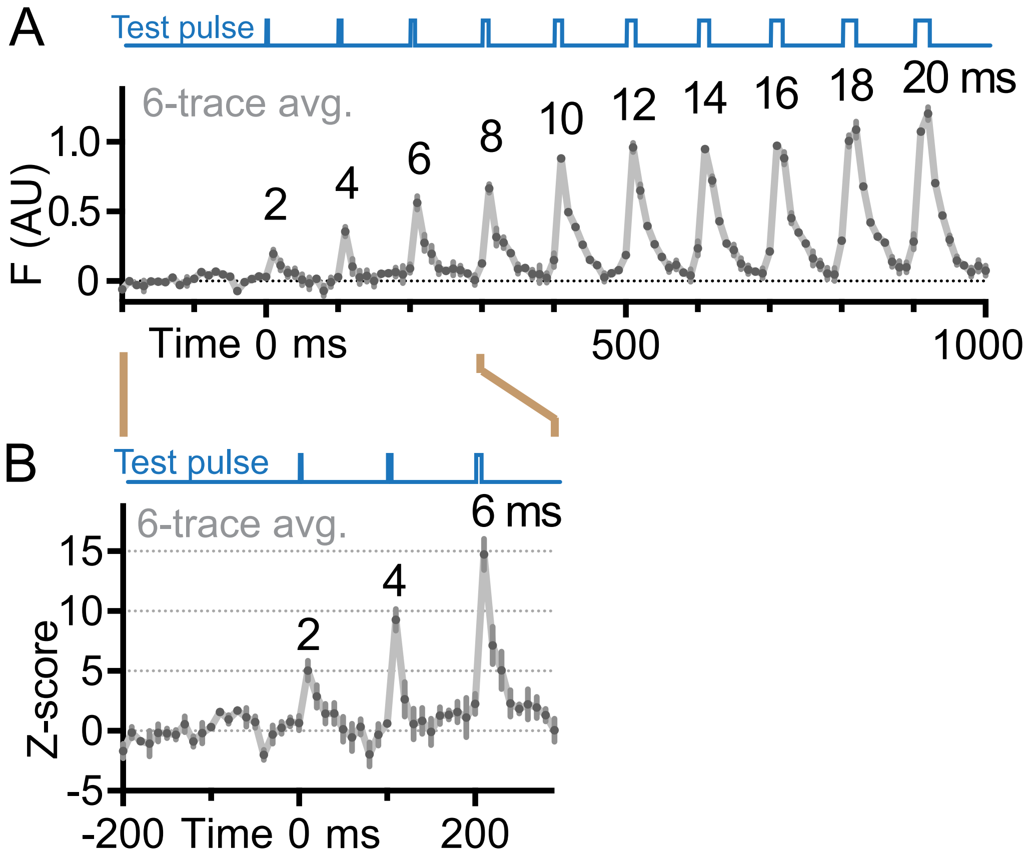

Fig. S6. Response of Orai1 Y80E-jGCaMP8f V203Y to short duration test pulses. (A and B) Orai1 Y80E-jGCaMP8f V203Y baseline-subtracted fluorescence response to a series of increasing duration test pulses to -100 mV; average of 6 total normalized traces from 3 cells. The same normalized and averaged fluorescence trace is plotted in (A) as Arbitrary Units (AU) and in (B) as the number of standard deviations (Z-score) from the -200 to 0 ms baseline. Test pulse duration is indicated above the plot, with a test pulse schematic above the plot in blue. Error bars are ± SEM.

Table S1

List of plasmid DNA constructs

Name	Reference
pGP-CMV-jGCaMP8f	(7)
pGP-CMV-jGCaMP8m	(7)
pGP-CMV-jGCaMP8s	(7)
Orai1-GCaMP6f	(2)
GGECO1.2-Orai1 Y80E	(2)
Orai1 Y80E-EGFP	This work
Flag-Orai1	(8)
EGFP-N1	Clontech
Orai1-EGFP	(2)
Orai1-jGCaMP8f	This work
Orai1-jGCaMP8m	This work
Orai1-jGCaMP8s	This work
Orai1 Y80E-jGCaMP8f	This work
Orai1-jGCaMP8f C48S	This work
Orai1-jGCaMP8f C70V	This work
Orai1-jGCaMP8f Q69M/C70V	This work
Orai1-jGCaMP8f V203T	This work
Orai1-jGCaMP8f V203Y	This work
Orai1 Y80E-jGCaMP8f V203Y	This work
pcDNA3(+)zeo-hSTIM1	(3)
Myc-STIM1	(9)
mCherry-STIM1	(4)
STIM1-EGFP	This work
STIM1-jGCaMP8f	This work
mCherry-STIM1-EGFP	This work
mCherry-STIM1-jGCaMP8f	This work
mCherry-STIM1-jGCaMP8f V203Y	This work

Table S2

List of DNA oligonucleotide primers

(SDM: Site-directed mutagenesis.)

Name	Technique	Sequence, 5' to 3'
G8FORC	PCR	CACAACCGGTCGCCACCCTGCACACGCGTCGCAAG
G8REVC	PCR	AGTCGCGGCCGCTTTAAAGCTTCGCTGTCATCATTTGTAC
ORAI1FORC	PCR	ACTCAGATCTCGAGCCACCATGCATCCGGAGCCCGCC
ORAI1REVC	PCR	TGCAGAATTCGGGCATAGTGGCTGCCGGGCGTC
IF-Fwd1	InFusion	GTCGCCACCCTGCACACGCGTCGCAAGAAGACCTTCAAG
IF-Rev1	InFusion	GTCACGAGGGTGGGCCAGGGCACGGGCAGCTTGCCGGTGGTGCAGATG
IF-C48SRev1	InFusion	GTCACGAGGGTGGGCCAGGGCACGGGCAGCTTGCCGGTGGTGCTGATG AACTTCAGG
IF-Fwd2	InFusion	CCCTGGCCCACCCTCGTGACCACCCTGACCTACGGCGTGCAGTGCTTC
IF-Q69M/C70V Fwd2	InFusion	CCCTGGCCCACCCTCGTGACCACCCTGACCTACGGCGTGATGGTCTTCA GCCGCTACC
IF-Rev2	InFusion	TATGATCTAGAGTCGCGGCCGCTTACTTCGCTGTCATCATTTG'
C70V Fwd	SDM	CCTACGGCGTGCAGGTCTTCAGCCGCTACC
C70V Rev	SDM	GGTAGCGGCTGAAGACCTGCACGCCGTAGG
V203T Fwd	SDM	GACAACCACTACCTGAGCACGGAGTCCAAACTTTCG
V203T Rev	SDM	CGAAAGTTTGGACTCCGTGCTCAGGTAGTGGTTGTC
V203Y Fwd	SDM	CCGACAACCACTACCTGAGCTACGAGTCCAAACTTTCGAAAG
V203Y Rev	SDM	CGAAAGTTTGGACTCCGTGCTCAGGTAGTGGTTGTC
S1FORC2	PCR	GGAAGAATTCGCCACCATGGATGTATGCGTCCGTC
S1REVC2	PCR	GTGTGGATCCCGCTTCTTAAGAGGCTTCTTAAAG

SI References

1. J. L. Dynes, A. V. Yeromin, M. D. Cahalan, Cell-wide mapping of Orai1 channel activity reveals functional heterogeneity in STIM1-Orai1 puncta. *J Gen Physiol* **152** (2020).
2. J. L. Dynes, A. Amcheslavsky, M. D. Cahalan, Genetically targeted single-channel optical recording reveals multiple Orai1 gating states and oscillations in calcium influx. *Proc Natl Acad Sci U S A* **113**, 440-445 (2016).
3. J. Roos *et al.*, STIM1, an essential and conserved component of store-operated Ca2+ channel function. *J Cell Biol* **169**, 435-445 (2005).
4. K. L. Ellefsen, J. L. Dynes, I. Parker, Spinning-Spot Shadowless TIRF Microscopy. *PLoS One* **10**, e0136055 (2015).
5. J. Schindelin *et al.*, Fiji: an open-source platform for biological-image analysis. *Nat Methods* **9**, 676-682 (2012).
6. C. A. Schneider, W. S. Rasband, K. W. Eliceiri, NIH Image to ImageJ: 25 years of image analysis. *Nat Methods* **9**, 671-675 (2012).
7. Y. Zhang *et al.*, Fast and sensitive GCaMP calcium indicators for imaging neural populations. *Nature* **615**, 884-891 (2023).
8. S. L. Zhang *et al.*, Store-dependent and -independent modes regulating Ca2+ release-activated Ca2+ channel activity of human Orai1 and Orai3. *J Biol Chem* **283**, 17662-17671 (2008).
9. M. I. Lioudyno *et al.*, Orai1 and STIM1 move to the immunological synapse and are up-regulated during T cell activation. *Proc Natl Acad Sci U S A* **105**, 2011-2016 (2008).
